# Endangered animals and plants are positively or neutrally related to wild boar (*Sus scrofa*) soil disturbance in urban grasslands

**DOI:** 10.1038/s41598-022-20964-4

**Published:** 2022-10-05

**Authors:** Valentin Cabon, Miriam Bùi, Henning Kühne, Birgit Seitz, Ingo Kowarik, Moritz von der Lippe, Sascha Buchholz

**Affiliations:** 1 Department of Ecology, Technische Universität Berlin, 12165 Berlin, Germany; 2 Berlin-Brandenburg Institute of Advanced Biodiversity Research (BBIB), 14195 Berlin, Germany; 3 Present Address: Université de Rennes 1, CNRS-ECOBIO (Ecosystèmes, biodiversité, évolution) UMR 6553, 35000 Rennes, France; 4 Present Address: Institute of Landscape Ecology, University of Münster, 48149 Münster, Germany

**Keywords:** Biodiversity, Community ecology, Conservation biology, Grassland ecology, Urban ecology, Ecology

## Abstract

Wild boar is increasingly establishing populations in the outskirts of European cities, with the largest German urban population occurring in Berlin. Related soil disturbance in grasslands is common and often considered as damage to biodiversity. However, it is unknown how animal and plant species in urban grasslands respond to wild boar activity - an important limitation for conservation management. We sampled plants, grasshoppers and sand lizards in 22 dry grasslands and measured wild boar activity. We show that plant diversity decreased with rooting intensity, but not species richness, endangered or specialist species. Relationships with animals were mostly positive. Grasshopper diversity, total richness and richness of endangered and specialist species were positively related to rooting, as was sand lizard abundance. These relationships contrast to mostly negative effects in the wild boar’s non-native range. This first multi-taxa study in a large city suggests that soil disturbance by wild boars is not necessarily a threat to biodiversity. An implication for conservation is to consider the context-dependence of biodiversity responses to wild boar activity. For dry grasslands, disturbed patches should be accepted in management plans rather than re-vegetated by seeding.

## Introduction

Cities can host a wide range of plant and animal species, including rare and threatened species^1–3^. Some native animal species increasingly colonize urban areas as “urban adapters” benefitting from additional resources in urban habitats^4–6^. The wild boar (*Sus scrofa*) is a prominent example of an urban adapter^7^. This species is native to Eurasia^8^ and increasingly extended its populations to the outskirts of European cities in the last decades^9,10^, with Berlin harbouring the largest urban population in Germany^11^. Urban populations are supposed to increase due to the high adaptability of the species^7,12^. Moreover, wild boars have become invasive globally^8^.

Wild boars are generally considered to be ecosystem engineers^13^ due to widespread soil disturbance in forests and other ecosystems. Rooting for foraging underground organs of plants, mushrooms or invertebrates is considered a key vector of environmental change^14,15^. It is easily identifiable by conspicuous soil excavation, which can reach up to thousands of square meters^16^.

Related environmental effects are multifarious, with contrasting biodiversity responses. Wild boars can support ecosystem functioning by enhancing ground aeration, maintaining open habitats, and creating niches for less competitive species^17–19^. However, wild boars are also associated with biodiversity losses and economic damage, making them be considered as a pest within their native range, or as invasive in the non-native range^20,21^. Increasing wild boar populations therefore pose a major management challenge^22,23^.

Most studies on wild boars’ impacts are from the non-native range, and many provide evidence of negative changes to native biodiversity and an often strong facilitation of biological invasions^8^. In the native range, previous research focussed on forests, with contrasting findings. For example, a positive effect of rooting on plant species richness was found in Sweden^24^, while Italian studies showed negative effects on species diversity but not on species richness^25,26^. In forests of the Netherlands, rooting impeded the regeneration of oak species^27^. In recently colonized taiga systems of Western Siberia, rooting was associated with a decreased species richness^28^.

Wild boar activities can alter animal communities as well^29^. Arthropods can be affected by predation^30^ or habitat modification^31^. In Spain, wild boars were found to feed on a wide range of arthropods, including *Coleoptera*, *Diptera* and *Orthoptera*^14^*.* Invertebrate richness was negatively related to wild boar abundance in another Spanish study^29^. Moreover, overgrazing, trampling or rooting could affect animal species sensitive to disturbance^29,32^. However, the creation of new microhabitats can also benefit endangered insect species^33^.

Conflicting results could depend on the covered biogeographic region, the local presence and activity of wild boars, the ecosystem type, the surveyed plant or animal taxa, or the observation period since initial disturbance. While environmental effects of wild boars have been mostly analysed in the non-native range, or in forests, the question of how rooting affects European grassland communities is critically understudied. Wild boars increasingly move within and beyond forests in European cities ^7,11,12^. How the increasing urban populations affect ecosystems of conservation concern outside forests, however, is an open question.

We chose dry grassland as a model system to analyse biodiversity-wild boar relationships. In Berlin, Germany, dry grasslands often occur within, or adjacent to forests, and are often subject to rooting. These extensively managed grasslands usually harbour many endangered species in urban settings^2,34,35^ and are legally protected according to Berlin’s Nature Conservation Act. To estimate the intensity of wild boar activity in dry grasslands we used two methods, i.e., direct observation by camera trap and indication by mapping of rooting traces. To capture effects of wild boar on different trophic levels, we used a multi-taxon approach, covering three taxa for which regional Red Lists are available (vascular plants, grasshoppers) or that are legally protected in the European Union, as is the sand lizard (*Lacerta agilis*). The latter is also a target species of nature conservation in Berlin and colonizes a range of urban habitats^36^.

In detail, we asked: (1) What is the best predictor of biodiversity responses in grassland related to wild boar activity, i.e. frequency data obtained by camera traps vs. amount of disturbed ground indicated by rooting traces? (2) How does wild boar activity relate to species richness and diversity in plants and grasshoppers, and to sand lizard abundance? (3) Do endangered species, habitat specialists and non-native species respond differently? (4) Does the composition of plant and grasshopper communities relate to the varying levels of wild boar activity?

## Methods

### Study area and study system

Study region was Berlin, the largest city of Germany, with a surface of 891 km^2^ and a population of 3.7 million inhabitants in 2021. About 59% of Berlin is developed with built-up areas and streets, while green and blue spaces cover 41%, including forests (18%) and grassland (5%)^37^. Grassland of conservation concern stretches over a range of near-natural and anthropogenic ecosystems^38^. According to a habitat suitability analysis, many natural and anthropogenic land use types are suitable for urban wild boar populations^12^.

**Figure 1:**
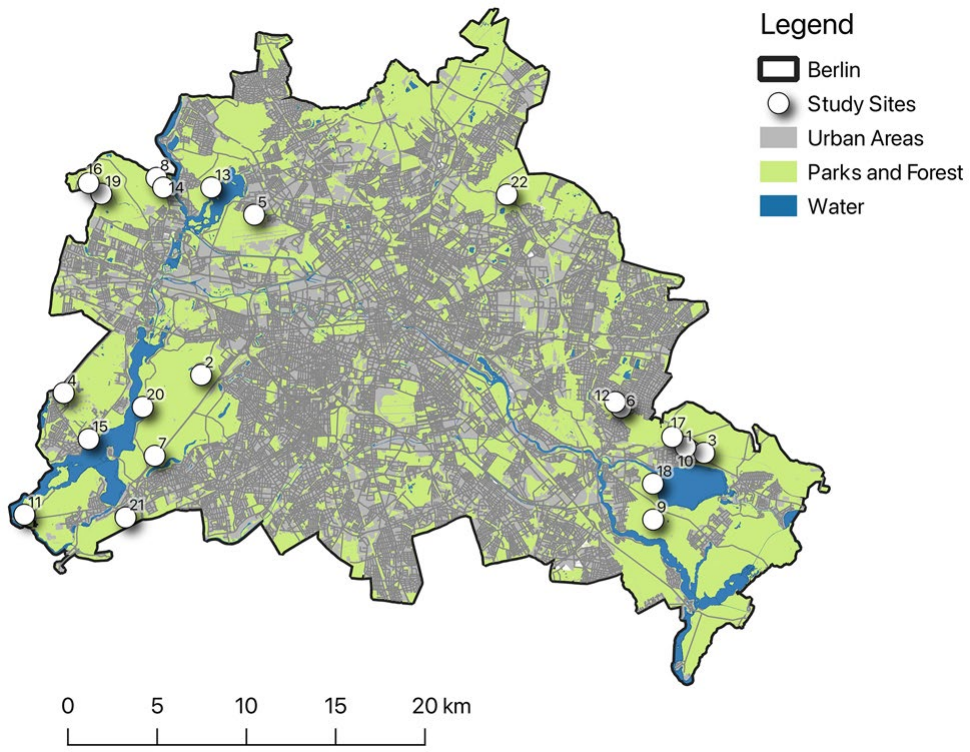
Location of the 22 study sites (white points) within Berlin. Developed areas are grey; forests, parks and other open spaces are green, waterbodies are blue. The map was created using ArcGIS ver. 10.3 (ESRI).

We selected 22 study sites from the dry grassland plots that have been established within the CityScapeLab Berlin, an experimental research platform to untangle urbanisation effects on biodiversity and biotic interactions^39^. These sites extend across the outskirts of Berlin and have developed on sandy soils in forest clearings or near forests (Fig. 1). They are extensively managed by mowing up to two times per year, without fertilization or irrigation. All patches belong to the type “dry grassland” according to the digital biotope maps of Berlin and Brandenburg^40^ and to the phytosociological vegetation type *Sedo-Scleranthetea*^41^.

### Wild boar activity sampling

To examine whether biodiversity changes are better predicted by the frequency of wild boar visits to grassland plots or by rooting traces, we applied two approaches: recording rooting traces as an indicator of wild boar activity and observation of the animals using camera traps.

We sampled rooting traces three times in two years (Sep. 2019, Mar. 2020 and Oct. 2020) to account for the history of disturbance^42^. At each site, we selected four 25-m transect lines based on randomly generated point grids, exactly located by GPS (Trimble® R10 receiver, TSC3 Controller) and following a standardised approach^43,44^. Two transect lines were oriented north–south and the remaining two west–east. We measured the length of the rooted and undisturbed ground within a buffer of one meter on each side of a measuring tape and distinguished fresh rooting from old rooting. We defined the former as open soil and the latter as re-vegetated rooting trace.

Other wild boar activities beyond rooting, like herbivory, predation or trampling likely depend on the frequency of visits to a site. To measure this frequency, we installed two camera traps (Secacam Wild-Vision Full HD 5.0) per study site. During three sampling periods of at least 20 days in 2020 (March to April, June to July and September to October), cameras with the same configuration were set at approximately 50 cm above ground surface. One camera was secured to a tree trunk or a wooden batten, five meters from the vegetation plot (see 2.3.1); the other was attached to a tree trunk at the edge of the dry grassland to reduce the chance of camera losses.

### Biodiversity sampling

In 2020, we conducted vegetation relevés on a 4 × 4 m plot at each of the 22 study sites. The plots were located in a rooted section of the above-described transect line, when rooting occured (Fig. S1). We chose this plot-based sampling rather than a transect sampling to restrict the assessment of rooting effects on vegetation that is directly impacted by rooting. We expected that otherwise the inclusion of several unrooted micro-habitats along the transect would have masked the actual decline of species sensitive to rooting. Each site was surveyed once between April and May and a second time in August. All vascular plants were recorded and species cover was visually estimated in 10% increments^45^. Total herb cover, litter cover and maximum height of herbs were sampled as relevant habitat features for the surveyed animal taxa. Data were stored and tabulated with the software TURBOVEG for Windows^46^.

We also distinguished between sub-groups as indicators of conservation value: (i) endangered plant species according to the regional Red List^47^ and (ii) dry grassland specialists. We defined dry grassland specialists as species that are assigned to the plant communities of the *Sedo-Scleranthetea*^48^. As a potential threat to biodiversity, we differentiated neophytes, i.e. non-native species introduced into the Berlin region after 1500^47^.

Along the same transects we used to record rooting activity, we surveyed grasshoppers acoustically for a total of 18 min. We also recorded grasshoppers’ stridulation (“singing”) with a recording device (Zoom H6 Handy Recorder) to allow retrospective verification of species determination. In a second step, we walked along the transects and collected silent grasshopper species by sweep netting. Each captured grasshopper was immediately released after identification. Transects were sampled in September 2019 and July 2020. We ensured for both periods that the weather conditions were warm and dry^49^. As for plants, we differentiated grasshoppers into dry grassland specialists^50^ and Red List species^51^. We did not differentiate any non-native grasshopper species although a range expansion as been observed in the last decades for *Calliptamus italicus*.

We surveyed sand lizard abundance at all study sites twice following a standard method^52^. The first survey took place from mid-April to mid-May 2020, and the second in June 2020, both during warm and dry weather. We sampled twice to cover the entire breeding season, when activity is highest and to limit the seasonal factor that could have strongly influenced the abundances in case of a single sampling session. During a period of 30-min, we walked across each site and counted each individual.

All biodiversity sampling methods were carried out in accordance with relevant local guidelines. No plant material was collected, and no animals were kept for experiments.

### Data analyses

We defined the intensity of rooting activity as the proportion of disturbed soil in the total length of the surveyed transects, calculated as the mean of three surveys per site. In addition to the total rooting, we also calculated intensities of fresh and old rooting activity.

We used the camera trap images to assess wild boar frequency independently from rooting activity. We extracted all images with animals using the program MegaDetector^53^. To assess wild boar frequency, we calculated the mean relative abundance index (RAI) from both cameras at each site, corresponding to the number of individuals depicted in images, divided by the number of days of camera exposure. Due to legal constraints and stolen material, three study sites were without camera traps.

All variables describing wild boar activity and habitat characteristics are listed in Table 1 and absence of collinearity was checked using Pearson or Spearman correlation (Table S1).

**Table 1:** Variables describing wild boar activities at study sites and habitat characteristics for 4 × 4 m vegetation plots.

		Unit	Mean ± SD	Range	Description
**Wild boar activities**					
Fresh rooting		%	7.318 ± 4.922	0–18.6	Signs of rooting visible as bare soil within a 1 m buffer along the transects
Old rooting		%	35.882 ± 25.663	0–86.77	Signs of rooting covered by new vegetation within a 1 m buffer along the transects
Total rooting		%	42.373 ± 26.481	0.4–100	Cumulated signs of fresh and old rooting
Occurence		Frequency	0.121 ± 0.109	0–0.4	Total abundances of wild boar sightings on each study site divided by overall days of camera trap exposure
**Habitat characteristics**					
Herb cover		%	54.864 ± 18.959	25–95	Proportion of herb coverage on vegetation plots
Height of herb layer		cm	14.091 ± 8.257	0–30	Height of herb layer on vegetation plots
Litter cover		%	17.455 ± 16.329	0–60	Proportion of litter coverage on vegetation plots

For vascular plants and grasshoppers, we calculated response variables as the overall species richness and Simpson diversity index. Then, we calculated species richness and relative abundance for red listed species, grassland specialists, and neophyte species. For sand lizards, we considered the sum of counted individuals.

We tested the response of all biodiversity variables to (1) total rooting and (2) wild boar frequency, using generalised linear mixed models (GLMM). We defined both wild boar variables as fixed effects and included the cover and height of the herb layer, and litter cover as random effects (Table 1). For each model, we calculated the coefficient of determination (R^2^) to assess the proportion of variation explained. We used AIC to compare the best models and therefore determined the most efficient predictor of relationships between wild boar rooting or frequency and response patterns in plants, grasshoppers, and sand lizards.

To explore how species composition of plant and grasshopper communities responds to wild boar activity and habitat features, we analysed relative species abundance datasets of both groups with multivariate methods, with the environmental variables shown in Table 1. To examine the variation in species composition between sites, we applied non-metric multidimensional scaling (NMDS) ordination, separately for plant and grasshopper communities. Prior to ordination, singletons were removed from both datasets, as they do not contribute to interpretable dissimilarities between sampling sites. Species abundances were log-transformed. NMDS ordination was based on the Bray–Curtis dissimilarity matrix. We used the ordination stress statistic as a measure of goodness of fit. To allow a visual representation of differences in species composition of plant and grasshopper communities at the study sites, we classified study sites into high and low rooting intensity classes, using the median as the cut-off value. To assess which local environmental parameters influence the community composition, we applied a permutational multivariate analysis of variance (PERMANOVA).

Where wild boar activities appeared as significantly related to species composition, we performed a partial redundancy analysis (partial-RDA) to assess the distribution of species along the gradient of wild boar activity (defined as a constrained axis). The other significant environmental variables were used as covariates. The explanatory power of rooting variables was evaluated with a Monte-Carlo test (999 permutations).

Additionally, we evaluated the fidelity of species to high and low rooting intensity classes using the IndVal (Indicator Value) procedure^55^ in the R library ‘indicspecies’^56^. The calculation of indicator values is based on the species abundance matrix and considers both relative abundances and frequencies of occurrence within the defined sample groups. The statistical significances of partial-RDA models and species indicator values were tested by Monte Carlo randomisation procedure (999 permutations).

All statistical and multivariate analyses were performed in R version 4.0.3^57^, using the packages ‘lme4’ for GLMMs computing^58^, ‘rsq’ for R^2^ calculation^59^, and ‘vegan’ for multivariate analysis^60^.

## Results

### Sampled species

We recorded a total of 160 vascular plant species (Table S2), with species richness varying from 16–43 per site (mean = 29.45; SD = 8.60). Most frequent species were the grasses *Agrostis capillaris*, *Festuca brevipila* and *Poa angustifolia*, and the herbs *Cerastium semidecandrum* and *Rumex acetosella*. Total species included 20 Red List species, 45 dry grassland specialists and 16 neophytes.

We sampled in total 2302 grasshopper individuals belonging to 24 species (Table S3), with species richness varying from 3–16 per site (mean = 10.27; SD = 3.57) and abundance varying from 22–216 individuals per site (mean = 104.64; SD = 48.34). The most frequent species were *Chorthippus mollis*, *Chorthippus brunneus*, *Chorthippus biguttulus* and *Pseudochorthippus parallelus* (Table S3). Eleven species were dry grassland specialists and 10 were red listed; both categories were strongly correlated (R = 0.979, *P* < 0.001).

We counted a total of 72 sand lizard individuals over the two sampling periods, with abundances ranging from 0 to 13 per site (mean = 3.27; SD = 3.94) (Table S4).

### Biodiversity measures related to wild boar rooting

Models built with rooting data were more efficient than the ones including frequency data (Table S5). We therefore focused on rooting in further analysis and used both the proportion of fresh and old rooting as fixed effects in all GLMMS.

**Figure 2:**
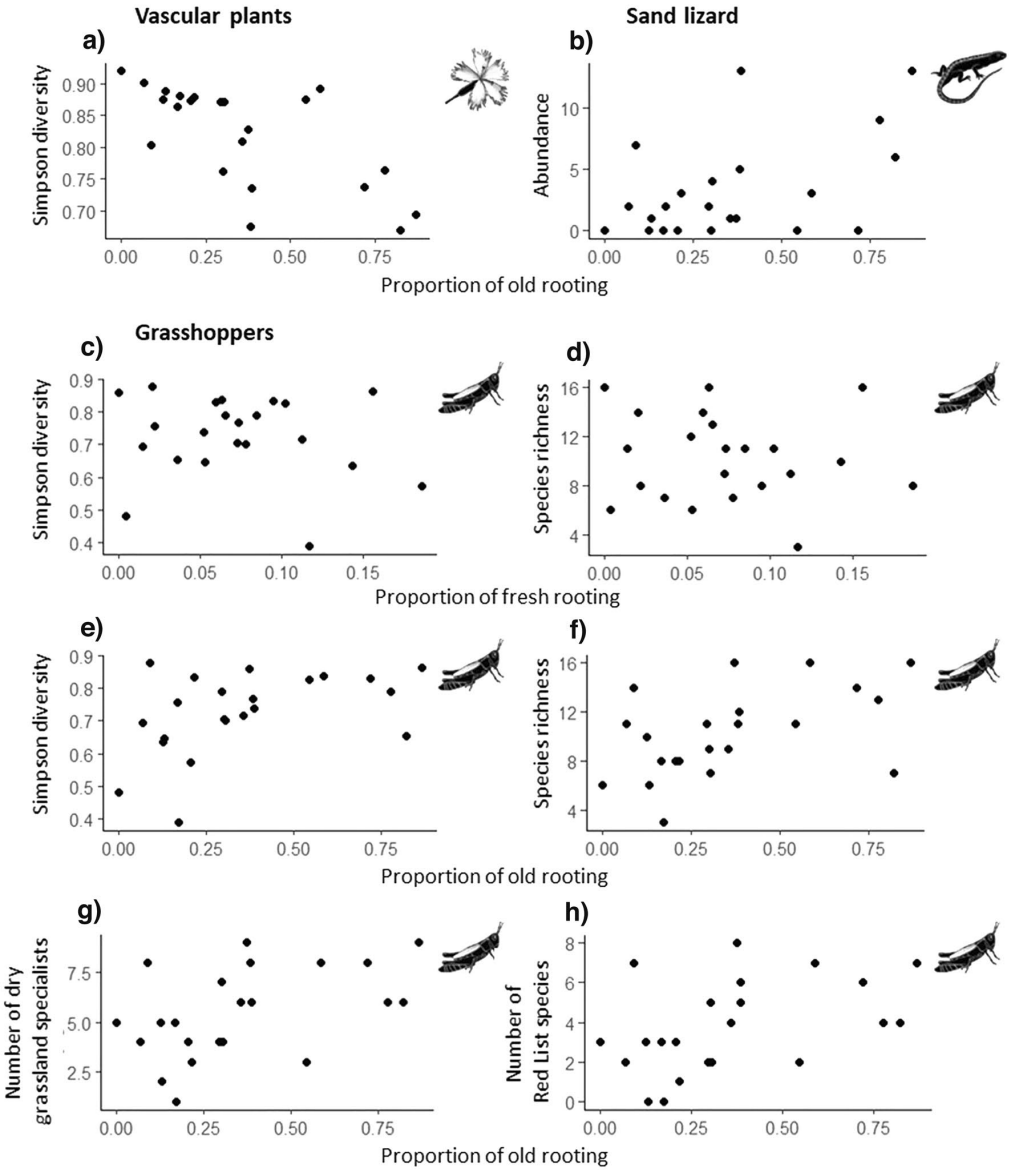
Observed significant relationships as revealed by GLMMs between biodiversity measures for plants, grasshoppers, and sand lizard abundance and wild boar rooting.

Old rooting was negatively related to plant species diversity (GLMM, *P* < 0.001, Est. = − 0.054, t = − 4.731, Fig. 2a), but not to plant species richness nor to the number or abundance of red list species, dry-grassland specialists or neophytes.

Diversity indices for grasshoppers were positively related to old rooting (GLMM, Simpson index: *P* = 0.002, Est. = 0.0817, t = 3.864, Fig. 2e; species richness: *P* = 0.005, Est. = 1.889, t = 3.555, Fig. 2f) and negatively to fresh rooting (GLMM, Simpson index: *P* = 0.037, Est. = − 0.045, t = − 2.370, Fig. 2c; species richness: *P* = 0.048, Est. = − 1.097, t = − 2.378, Fig. 2d). Richness of dry grassland specialists (GLMM, *P* = 0.020, Est. = 1.010, t = 2.550, Fig. 2g) and of Red List species (GLMM, *P* = 0.036, Est. = 0.959, t = 2.268, Fig. 2h) were positively related to old rooting.

Sand lizard abundance was positively linked to old rooting as well (GLMM, *P* = 0.006, Est. = 0.733, z = 2.731, Fig. 2b). All GLMM results are presented in Table S6.

### Community composition related to wild boar rooting

**Figure 3:**
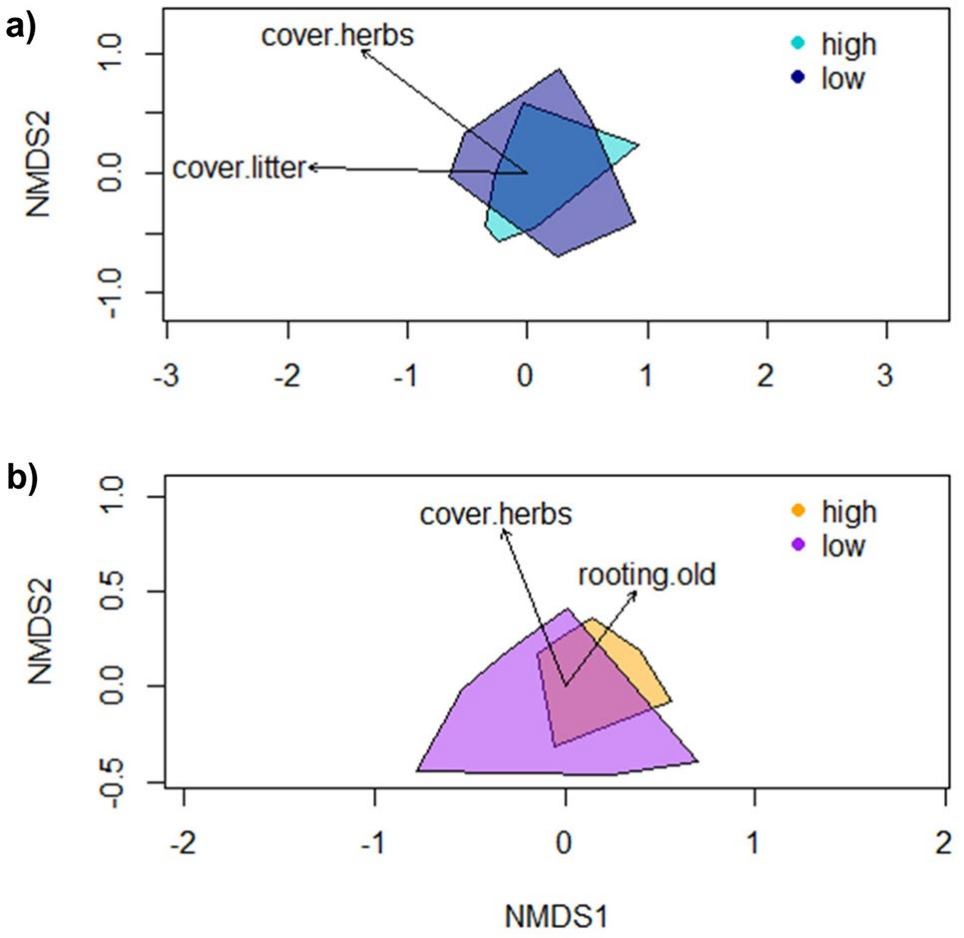
Results of NMDS displaying environmental variables and similarities in species composition of (**a**) vascular plants and (**b**) grasshoppers in high and low rooting levels. Cover of herbs and cover of litter significantly explained the species composition of vascular plants, whereas cover of herbs and the proportion of old rooting explained the species composition of grasshoppers.

Plant community composition was significantly related to herb cover (*P* = 0.009; R^2^ = 0.088) and litter cover (*P* = 0.041; R^2^ = 0.073) but not to any of the wild boar variables (NMDS, stress = 0.199, Fig. 3a).

In contrast, grasshopper communities were significantly structured by the proportion of old rooting traces (*P* = 0.042; R^2^ = 0.082) and herb cover (*P* = 0.001; R^2^ = 0.124) (NMDS, stress = 0.190, Fig. 3b). The proportion of old rooting traces appeared significant as well, when tested by partial-RDA (F = 1.941, *P* = 0.020). The first axis explained 9.27% of data variability in RDA and 19.97% in PCA. Four species were associated with high rooting intensity (*Myrmeleotettix maculatus*, *Calliptamus italicus, Tettigonia viridissima, Platycleis albopunctata*) and another four with low rooting intensity (*Chorthippus biguttulus*, *Leptophyes punctatissima*, *Decticus verrucivorus, Pseudochorthippus parallelus*; Fig. S2, Table S7). With regard to species scores obtained by partial-RDA (Fig. S2, Table S7), three red listed species were associated with a high proportion of old rooting traces. Among these, *Calliptamus italicus* is considered extinct in Berlin (category 0) and is protected by law, *Platycleis albopunctata* and *Myrmeleotettix maculatus* are considered near threatened (category V). The latter obtained a significant species indicator value for being associated with high proportions of old rooting (*P* = 0.009).

## Discussion

Wild boars are increasingly present in the outskirts of European cities such as within the borders of Berlin. While these animals are usually seen through a lens of the damage they can cause^23,61^, empirical studies on biodiversity impacts in urban regions are missing thus far.

This is the first multi-taxon study of how wild boar activity relates to biodiversity in dry grassland, an ecosystem type of conservation concern in Berlin and beyond^34,35,38^. Assessing wild boar activity with two approaches (camera traps, rooting traces) revealed that sampling of rooting traces best predicted biodiversity measures. We thus based further analyses on this.

As a major insight we found that wild boar activity was differently related to biodiversity measures of plants, grasshoppers and sand lizards. These relationships were multidirectional but mostly positive. Responses in endangered species were positive in animals and neutral in plants. This has important implications for conservation strategies that have so far addressed wild boar activities primarily as a threat to dry grassland diversity.

Disturbance by wild boars is an important driver of vegetation change^62,63^, which has been shown for wild boars particularly in their non-native range^8^. In our study, old rooting was negatively related only to Simpson diversity of plants, while we could not detect any effect on plant species richness, red list species, neophytes or community composition. This suggests that rooting leads to changes in dominance of plant species but not to a replacement of plant species in dry grassland. We explain missing relationships to fresh rooting with legacy effects. In the year of disturbance (i.e. fresh rooting), the relative abundance of plant species in the vegetation plot did not change significantly. In the following years, Simpson diversity decreased. This is to be expected if annual pioneer species (i.e. therophytes) that were already present before remained present directly after disturbance due to improved germination conditions, but declined in abundance with time. This could be explained by the continuous degradation of germination conditions after disturbance due to the increased abundance of perennial species (e.g. grass species building rhizomes). The subsequently decreasing availability of bare soil and light at the ground reduces regeneration niches of pioneer species which can explain the observed decrease in biodiversity. As we intentionally restricted vegetation sampling to directly rooted sampling plots, the effects of rooting on vegetation could partly be mediated by the continuity of small unrooted micro-habitats within the habitat mosaic of the sampling sites. In these unrooted remnants, species sensitive to rooting may have persisted even after heavy rooting of the entire sampling sites. It remains thus an open question whether the diversity of the rooted plots may recover due to recolonization from unrooted remnants in their vicinity.

Our results differ from studies in the non-native range of wild boars, which showed far-reaching effects on plant assemblages, including a facilitation of plant invasions^8^. In California, an increase in species richness after disturbance was found, particularly in non-native species^64^. In temperate drylands of Argentina, a decrease in both diversity and richness of plants was found^63^, yet with a long-term recovery of vegetation after exclusion of wild boars^65^. Moreover, different types of plants responded differently to rooting in these studies.

Divergent from many studies in the non-native range^20,66,67^, wild boars in our study do not appear to foster plant invasions in dry grassland. In contrast, dog walking in the same study area (Berlin) and the same dry grasslands was positively related to non-native plant richness^68^. While both wild boars and dogs are vectors of plant dispersal^69,70^, the dispersal of non-native plants into dry grasslands by dogs appears to be more effective, likely due to dogs being more exposed to non-native seed sources in developed urban areas.

In Berlin, rooting is common in forests and adjacent green spaces, and wild boars are addressed as a threat to endangered plant species^47^. While population survival of endangered plant species is critically challenged in Berlin forests and grasslands^71^ we found no evidence of negative impacts of wild boars on red listed plant species. However, local extinction of very small populations cannot be excluded if destroyed by wild boars.

As for plants, old rooting was a significant predictor of biodiversity measures in the surveyed animal groups, with prevailing positive relationships. Only fresh rooting was negatively related to grasshopper diversity and richness. However, this effect has been reversed over time since old rooting was positively related to grasshopper diversity and richness as well as to dry grassland specialists and Red List species. In the same vein, old rooting was positively associated with sand lizard abundance.

Grasshoppers are generally sensitive to changes in grassland environments. Modifications of the vegetation structure^72^ or soil disturbance shape micro-niches and modulate the microclimate^29,73^. Accordingly, grasshopper communities in our study were structured along the intensity of old rooting (Fig. S2), and species were differently associated with low or high intensity rooting. The latter include the near threatened *Myrmeleotettix maculatus,* which depends on scarce vegetation and bare ground^73^.

Our results suggest temporal variation in grasshoppers’ responses to rooting. The shift from a negative relationship of biodiversity measures with fresh rooting to a positive relationship with old rooting is likely due to structural changes in the grassland habitats. Re-vegetated old rooting traces appear to be beneficial for most grasshopper species - albeit not for all. Consistently, the rare steppe specialist *Stenobothrus eurasius* in Pannonian dry grassland is negatively affected by wild boar activity^31^.

Our findings indicate a beneficial role of wild boars for grasshopper conservation in Berlin. Here, local disturbance by rooting likely promotes niche differentiation in previously homogeneously structured grasslands, thereby increasing the number and diversity of endangered grasshopper species and dry grassland specialists.

Analogously, the abundance of sand lizards was positively related to old rooting intensity. Sand lizards require structurally complex habitats, with a combination of sites for thermoregulation, foraging, hiding, hibernation, and oviposition^74^. Decreasing microhabitat heterogeneity, in contrast, leads to a decline in abundance^75^. Our study suggests that wild boar rooting enhances sand lizard populations in dry grasslands, likely by creating a mosaic of bare ground, litter, sparse and dense vegetation.

The prevailing positive effects of wild boars on grasshoppers and sand lizards become understandable against the background of forest history in Berlin^76^: Many forests, with interspersed or adjacent grasslands, were grazed until the middle of the nineteenth century, which was associated with soil disturbance. Disturbances also occurred through clearing and reforestation after World War II. In recent decades, forests have become predominantly denser; open grassland areas have often become overgrown. The subsequently reduced environmental heterogeneity is a threat to open-grassland species^76^. Soil disturbance by wild boars could compensate for the loss of disturbance due to the cessation of grazing of forest-grassland complexes, thereby supporting grasshopper species and sand lizards.

## Conclusions

This first study of effects of wild boars on the biodiversity of dry grasslands in a Central European metropolitan area suggests that this ecosystem type is relatively resilient to local disturbances by rooting. Negative impacts on vascular plants were limited, whereas grasshopper species and sand lizards were predominantly enhanced, including endangered species. These results differ greatly from studies from the non-native range of wild boars and highlight that the impacts of this globally distributed ecosystem engineer highly depend on the biogeographic context.

Forest-grassland complexes in many European landscapes are historically adapted to ground disturbance by grazing, and rooting activity of wild boars could compensate for the loss of habitat heterogeneity, which has declined since the end of historic forest grazing. Although negative consequences for grassland biodiversity cannot be ruled out with higher densities of wild boar, our study suggests that rooting appears to be compatible with endangered plant species and can support endangered animal species. Conservation managers should therefore refrain from re-vegetating rooting traces as soon as possible, e.g., with seeding, but instead consider the open or sparsely re-vegetated traces as a beneficial component of site heterogeneity.

This sheds a positive light on ground disturbance by wild boars. Certainly, rooting can cause undesirable damage when landscaped green spaces are disturbed. Also, the occasionally required mowing of semi-natural dry grasslands may be impeded when severe soil elevations must be levelled beforehand. Our main conclusion is that conspicuous ground disturbance by wild boars may not always be assessed as ecological damage, but may well be accepted as natural disturbance in management strategies to promote biodiversity and endangered species in grassland.

## Supplementary Information

Supplementary Information.

## Data Availability

Data and R-codes are available from the following repository: https://github.com/vcabon/WildBoarGrasslands.
