# Supplementary material for: Endangered animals and plants are positively or neutrally related to wild boar (Sus scrofa) soil disturbance in urban grasslands
Source: Sci Rep. 2022 Oct 5;12:16649. doi: 10.1038/s41598-022-20964-4 (PMC9534874; doi:10.1038/s41598-022-20964-4)
Supplement: Supplementary file 1 — Supplementary Information. [file 41598_2022_20964_MOESM1_ESM.docx]

**Supplementary Information for “Endangered animals and plants are positively or neutrally related to wild boar (Sus scrofa) soil disturbance in urban grasslands”**

Valentin Cabon^1,2^, Miriam Bùi^1^, Henning Kühne^1^, Birgit Seitz^1,3^, Ingo Kowarik^1,3^, Moritz von der Lippe^1,3^, Sascha Buchholz^1,3,4^

^1^ Technische Universität Berlin, Department of Ecology, D-12165 Berlin, Germany

^2^ new address: Université de Rennes 1, CNRS-ECOBIO (Ecosystèmes, biodiversité, évolution) UMR 6553, 35000 Rennes, France

^3^ Berlin-Brandenburg Institute of Advanced Biodiversity Research (BBIB), D-14195 Berlin, Germany

^4^ new address: Institute of Landscape Ecology, University of Münster, D-48149 Münster, Germany

**Contents:**

**Figure S1:** Examples of 100-meter transects (4x 25 m, in yellow) for sampling of rooting traces.

**Figure S2:** Ordination plot displaying the results of the partial-RDA.

**Table S1**: Matrix of correlations among predictor variables.

**Table S2:** Abundances of vascular plant species recorded in Mai and August 2020.

**Table S3:** Abundances and status of grasshopper species recorded in September 2019 and July 2020.

**Table S4:** Abundances of sand lizards.

**Table S5:** Comparison of AIC values.

**Table S6:** GLMM summaries.

**Table S7:** Species scores obtained by partial-RDA applied on grasshopper data.


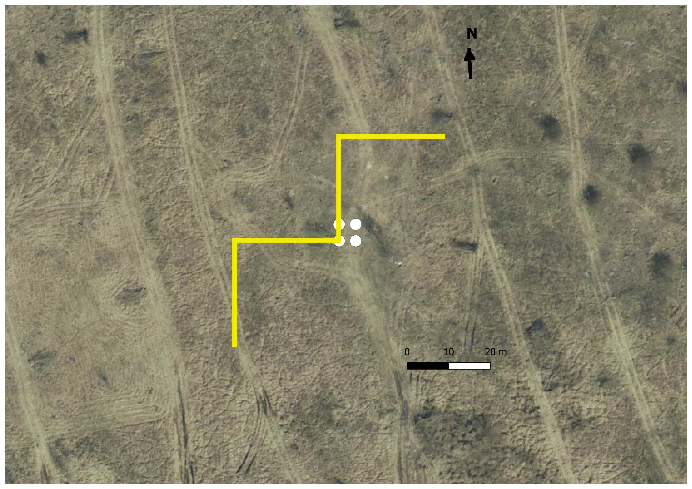

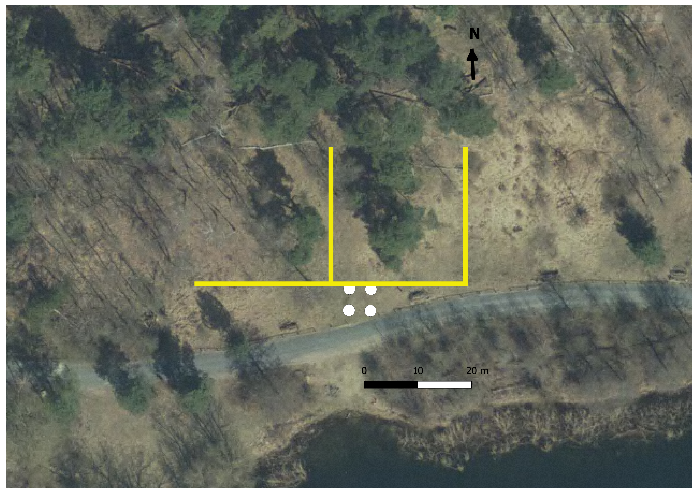


**Fig. S1** Examples of 100-meter transects (4x 25 m, in yellow) for sampling of rooting traces. The white dots represent the plots for the vegetation relevés. Maps were generated using QGIS 2.14 (https://download.qgis.org/downloads/)


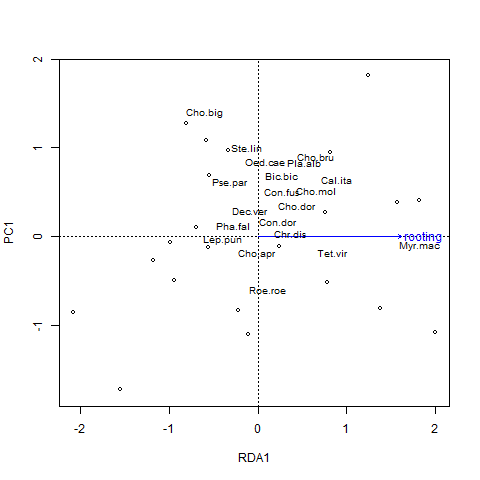


**Fig. S2** Ordination plot displaying the results of the partial-RDA. Grasshopper species significantly assembled along the old rooting intensity (RDA1; P = 0.022). Study site are represented with circles. Only species occurring at more than one study site are displayed. Abbreviations: Bic.bic = *Bicolorana bicolor*, Cal.ita = *Calliptamus italicus*, Cho.apr = *Chorthippus apricarius*, Cho.big = *Chorthippus biguttulus*, Cho.bru = *Chorthippus brunneus*, Cho.dor = *Chorthippus dorsatus*, Cho.mol = *Chorthippus mollis*, Chr.dis = *Chrysochraon dispar*, Con.fus = *Conocephalus fuscus*, Con.dor = *Conocephalus dorsalis*, Dec.vec = *Decticus verrucivorus*, Lep.pun = *Leptophyes punctatissima*, Myr.mac = *Myrmeleotettix maculatus*, Oed.cae = *Oedipoda caerulescens*, Pha.fal = *Phaneroptera falcata*, Pla.alb = *Platycleis albopunctata*, Pse.par = *Pseudochorthippus parallelus*, Roe.roe = *Roeseliana roeselii*, Ste.lin = *Stenobothrus lineatus*, Tet.vir = *Tettigonia viridissima*.

| **Table S1** Matrix of correlations among predictor variables (Pearson correlations (R), Spearman correlations (Rs), p-values (P)). | | | | | | | |
| --- | --- | --- | --- | --- | --- | --- | --- |
|  | Total rooting | Fresh rooting | Old rooting | Frequence wild boar | Cover herb | Cover litter | Height herbs |
| Total rooting |  | R = 0.323 | R = 0.975 | Rs = 0.362 | R = 0.143 | Rs = -0.266 | R = -0.510 |
|  |  | P = 0.142 | P < 0.001 | P = 0.098 | P = 0.526 | P = 0.231 | P = 0.015 |
| Fresh rooting | R = 0.323 |  | R = 0.161 | Rs = -0.113 | R = -0.001 | Rs = 0.110 | R = 0.076 |
|  | P = 0.142 |  | P = 0.473 | P = 0.618 | P = 0.996 | P = 0.624 | P = 0.738 |
| Old rooting | R = 0.975 | R = 0.161 |  | Rs = 0.426 | R = 0.157 | Rs = -0.275 | R = -0.517 |
|  | P < 0.001 | P = 0.473 |  | P = 0.048 | P = 0.486 | P = 0.215 | P = 0.014 |
| Frequence wild boar | Rs = 0.362 | Rs = -0.11 | Rs = 0.426 |  | Rs = 0.077 | Rs = -0.171 | Rs = -0.33 |
|  | P = 0.098 | P = 0.618 | P = 0.048 |  | P = 0.734 | P = 0.446 | P = 0.128 |
| Cover herb | R = 0.143 | R = -0.001 | R = 0.157 | Rs = 0.077 |  | Rs = 0.126 | R = -0.011 |
|  | P = 0.526 | P = 0.996 | P = 0.486 | P = 0.734 |  | P = 0.578 | P = 0.960 |
| Cover litter | Rs = -0.26 | Rs = 0.111 | Rs = -0.27 | Rs = -0.171 | Rs = 0.126 |  | Rs = 0.359 |
|  | P = 0.231 | P = 0.624 | P = 0.215 | P = 0.446 | P = 0.578 |  | P = 0.101 |
| Height herbs | R = -0.510 | R = 0.076 | R = -0.517 | Rs = -0.335 | R = -0.011 | Rs = 0.360 |  |
|  | P = 0.015 | P = 0.738 | P = 0.014 | P = 0.128 | P = 0.960 | P = 0.101 |  |

| **Table S2** Abundances of vascular plant species recorded in Mai and August 2020. DGS = Dry grassland specialists (Jäger, 2016), N = Neophytes (Jäger, 2016), RL = Red List species (Seitz et al., 2018): *= not endangered, 2 = endangered, 3 = vulnerable, V = near threatened). The identification of the species followed Jäger (2016), the taxonomy Buttler and Thieme (2017). | | | | | | | | | | | | | | | | | | | | | | | | | |
| --- | --- | --- | --- | --- | --- | --- | --- | --- | --- | --- | --- | --- | --- | --- | --- | --- | --- | --- | --- | --- | --- | --- | --- | --- | --- |
| **Species** | **1** | **2** | **3** | **4** | **5** | **6** | **7** | **8** | **9** | **10** | **11** | **12** | **13** | **14** | **15** | **16** | **17** | **18** | **19** | **20** | **21** | **22** | **DGS** | **N** | **RL** |
| *Acer platanoides* | . | . | . | . | 0.1 | . | . | . | . | . | . | . | . | . | 0.1 | . | . | . | . | . | . | . | . | X | * |
| *Achillea millefolium* | 2 | 1 | 2 | 0.1 | 4 | 0.1 | 1 | 0.1 | . | 1 | . | 1 | 20 | . | . | 2 | 1 | . | . | . | 1 | . | . | . | * |
| *Agrimonia eupatoria* | . | . | . | . | . | . | . | . | . | . | . | 0.1 | . | . | . | . | . | . | . | . | . | . | . | . | * |
| *Agrostis capillaris* | . | 1 | 2 | . | 1 | . | 1 | 10 | . | 2 | 1 | 1 | 20 | 20 | 2 | 2 | 2 | 2 | 2 | 1 | . | . | X | . | * |
| *Agrostis stolonifera* | . | . | . | . | . | . | . | . | . | . | . | . | . | . | . | . | . | . | . | . | 2 | . | . | . | * |
| *Ajuga genevensis* | . | . | . | . | . | . | . | . | . | . | . | . | . | . | . | . | . | . | . | . | 0.1 | . | X | . | 3 |
| *Alliaria petiolata* | . | . | 0.1 | . | . | . | . | . | . | . | . | . | . | . | . | . | . | . | . | . | . | . | . | . | * |
| *Anchusa officinalis* | . | . | . | . | . | . | . | . | . | . | . | . | . | . | 0.1 | . | . | . | . | . | . | . | . | . | * |
| *Anthoxanthum odoratum* | . | . | . | . | . | . | . | . | . | 2 | . | . | 0.1 | . | . | . | . | 1 | . | . | . | . | . | . | * |
| *Arabidopsis thaliana* | . | . | 1 | 0.1 | 0.1 | . | 1 | . | . | . | . | . | 1 | 1 | 1 | . | 0.1 | 1 | . | 1 | 1 | . | . | . | * |
| *Arenaria serpyllifolia* | . | 1 | . | 1 | 0.1 | 0.1 | 1 | . | 1 | . | . | 1 | 1 | 0.1 | . | . | 1 | 1 | 0.1 | 1 | 1 | 0.1 | . | . | * |
| *Armeria maritima* subsp. *elongata* | . | . | . | . | . | . | . | . | . | . | . | 1 | . | . | 4 | . | . | . | 1 | . | . | . | X | . | V |
| *Arrhenatherum elatius* | . | . | . | . | . | . | . | . | . | . | 0.1 | . | 20 | . | . | 1 | . | . | . | . | . | . | . | X | * |
| *Artemisia campestris* | 0.1 | . | . | 2 | . | 4 | . | . | 4 | 0.1 | . | . | . | 0.1 | 0.1 | . | . | 1 | . | 2 | . | 1 | X | . | * |
| *Artemisia vulgaris* | 0.1 | . | 1 | . | 0.1 | . | . | . | . | . | . | . | . | . | . | . | . | . | . | . | . | . | . | . | * |
| *Berteroa incana* | 1 | . | 1 | 1 | 1 | 1 | 1 | . | . | 0.1 | 1 | 1 | . | . | 1 | . | 1 | 1 | . | 1 | 1 | 0.1 | . | X | * |
| *Bromus hordeaceus* | 2 | . | . | . | . | . | . | . | 1 | . | . | 1 | 1 | . | . | . | 10 | . | 4 | 1 | . | . | . | . | * |
| *Bromus inermis* | . | . | . | 1 | . | . | . | . | . | . | . | . | . | . | . | . | . | . | . | 20 | . | . | . | X | * |
| *Bromus sterilis* | . | . | . | . | 10 | . | 1 | . | . | . | . | . | . | . | . | . | . | . | . | 1 | . | 1 | . | . | * |
| *Calamagrostis epigejos* | 1 | . | 1 | 2 | 4 | 1 | . | . | 2 | 1 | . | 2 | . | . | . | 1 | . | 2 | 10 | 2 | 10 | . | . | . | * |
| *Capsella bursa-pastoris* | 1 | 0.1 | 1 | . | . | . | . | . | . | . | . | . | . | . | . | . | 0.1 | . | . | . | . | . | . | . | * |
| *Cardamine hirsuta* | . | . | . | . | 0.1 | . | . | . | . | . | . | . | 1 | . | 1 | . | . | . | . | . | . | . | . | X | * |
| *Carex arenaria* | . | . | . | . | . | . | 10 | . | . | . | . | . | . | . | . | . | . | 2 | . | . | . | . | X | . | * |
| *Carex caryophyllea* | . | . | . | . | . | . | . | . | . | 4 | . | . | . | . | . | . | . | . | . | . | . | . | X | . | 2 |
| *Carex hirta* | 10 | . | 10 | . | 10 | 2 | 0.1 | . | . | 2 | . | 20 | . | . | . | . | 10 | 1 | . | . | 10 | . | . | . | * |
| *Carex ligerica* | . | . | . | . | . | . | . | . | . | 1 | . | . | . | . | . | . | . | . | . | . | . | . | X | . | V |
| **Species** | **1** | **2** | **3** | **4** | **5** | **6** | **7** | **8** | **9** | **10** | **11** | **12** | **13** | **14** | **15** | **16** | **17** | **18** | **19** | **20** | **21** | **22** | **DGS** | **N** | **RL** |
| *Carex praecox* | . | . | . | . | 1 | . | . | . | . | . | 30 | . | . | . | 4 | . | . | . | . | 1 | 10 | . | X | . | * |
| *Centaurea australis* | . | . | . | . | . | . | 0.1 | . | . | . | . | . | . | . | . | . | . | . | . | . | . | . | . | X | * |
| *Centaurea jacea* | . | . | . | . | . | . | . | . | . | . | . | 10 | . | . | . | . | . | . | . | . | . | . | . | . | V |
| *Centaurea stoebe* | 1 | . | . | 0.1 | . | 1 | . | . | 1 | . | . | . | . | . | . | . | 1 | 1 | . | . | . | . | X | . | * |
| *Cerastium arvense* | . | . | 1 | . | . | . | . | . | . | 1 | 1 | . | . | . | . | . | . | . | . | . | . | . | X | . | * |
| *Cerastium semidecandrum* | 1 | 10 | . | 2 | 1 | 4 | 10 | 1 | 1 | . | 1 | 1 | 4 | 2 | 4 | 1 | 10 | 4 | 10 | 10 | 1 | 1 | X | . | * |
| *Chenopodium album* | . | . | . | . | 1 | . | . | . | . | . | . | . | . | . | . | . | 0.1 | . | . | . | . | . | . | . | * |
| *Chondrilla juncea* | . | 0.1 | . | . | . | . | . | . | . | . | . | . | . | . | . | . | . | 0.1 | . | . | . | 1 | X | . | * |
| *Convolvulus arvensis* | . | . | . | . | . | 1 | 1 | . | . | . | 4 | . | 2 | . | . | . | 2 | . | . | . | . | . | . | . | * |
| *Conyza canadensis* | . | 0.1 | . | . | . | . | 1 | . | . | . | . | 0.1 | . | 0.1 | . | 1 | 1 | 0.1 | . | 0.1 | . | . | . | X | * |
| *Corynephorus canescens* | . | . | . | . | . | 10 | . | 1 | . | . | . | . | . | 10 | . | . | . | . | . | . | . | 2 | X | . | * |
| *Crataegus monogyna* agg. | . | . | . | . | . | . | . | . | . | . | . | . | . | . | . | . | . | . | 0.1 | . | . | . | . | . | * |
| *Crepis capillaris* | . | 0.1 | . | . | . | . | . | . | . | . | 0.1 | 1 | 1 | . | . | . | 0.1 | . | 2 | . | 1 | . | . | . | * |
| *Cytisus scoparius* | . | . | . | . | . | . | . | . | . | . | . | . | . | 1 | . | . | . | . | . | . | . | . | . | . | * |
| *Dactylis glomerata* | . | . | . | . | . | . | . | . | . | . | . | 1 | . | . | . | . | . | . | . | . | 2 | . | . | . | * |
| *Daucus carota* | . | . | . | 0.1 | . | . | . | . | . | 0.1 | . | . | . | . | . | . | . | . | . | . | . | 0.1 | . | . | * |
| *Dianthus deltoides* | . | . | . | . | . | . | . | . | . | . | 0.1 | 2 | 20 | . | . | 2 | . | . | . | . | 1 | . | X | . | 3 |
| *Digitaria ischaemum* | . | . | . | . | . | . | . | 2 | . | . | . | . | . | 2 | . | . | . | . | . | . | . | . | . | . | * |
| *Draba verna* | . | 1 | . | . | . | . | 0.1 | . | 1 | . | . | . | . | . | 1 | . | . | 1 | 1 | 0.1 | . | . | X | . | * |
| *Echium vulgare* | . | . | 0.1 | . | . | 0.1 | 1 | . | . | 0.1 | . | . | . | . | . | . | 0.1 | . | . | 0.1 | . | . | X | . | * |
| *Elymus repens* | 1 | 2 | 20 | . | 1 | 2 | 1 | 2 | . | 2 | 1 | 0.1 | . | . | 1 | . | 20 | 1 | . | 1 | . | . | . | . | * |
| *Equisetum x moorei* | . | . | . | . | . | . | . | . | . | . | . | . | . | . | . | . | . | . | . | . | 0.1 | . | . | . | 2 |
| *Eragrostis minor* | . | . | . | . | . | 1 | . | . | . | . | . | . | . | . | . | . | . | . | . | . | . | . | . | X | * |
| *Erigeron acris* | . | . | . | 1 | . | . | . | . | . | . | . | . | . | . | . | . | . | . | . | . | . | . | . | . | V |
| *Erodium cicutarium* | . | . | . | . | . | . | . | . | 0.1 | . | . | . | . | . | . | . | 0.1 | . | . | 1 | . | . | . | . | * |
| *Euonymus europaea* | . | . | . | . | . | . | . | . | . | 2 | . | . | . | . | . | . | . | . | . | . | . | . | . | . | * |
| *Euphorbia cyparissias* | 0.1 | . | 1 | . | 4 | 1 | . | . | 1 | 0.1 | . | . | . | . | 1 | . | 1 | 2 | . | 1 | 1 | . | X | . | * |
| *Fagus sylvatica* | . | . | . | . | . | . | . | . | . | . | . | . | . | . | . | . | . | 0.1 | . | . | . | . | . | . | * |
| *Fallopia convolvulus* | . | . | 0.1 | . | . | . | . | . | . | . | . | . | . | . | . | . | . | . | . | . | . | . | . | . | * |
| *Festuca brevipila* | 1 | 30 | 2 | 30 | . | . | 40 | 20 | 10 | 20 | 20 | . | . | . | 40 | 2 | . | 30 | . | 2 | 20 | 1 | X | . | * |
| **Species** | **1** | **2** | **3** | **4** | **5** | **6** | **7** | **8** | **9** | **10** | **11** | **12** | **13** | **14** | **15** | **16** | **17** | **18** | **19** | **20** | **21** | **22** | **DGS** | **N** | **RL** |
| *Festuca ovina* | . | . | . | . | . | . | . | . | . | . | . | . | . | . | . | 30 | . | . | . | . | . | . | X | . | * |
| *Festuca rubra* agg. | 1 | . | 10 | 10 | . | 20 | . | 1 | . | 10 | 2 | 10 | 10 | . | . | . | 1 | . | . | . | 2 | . | . | . | * |
| *Galium boreale* | . | . | . | . | . | . | . | . | . | 1 | . | . | . | . | . | . | . | . | . | . | . | . | . | . | 3 |
| *Galium mollugo* | . | . | . | 0.1 | . | . | . | . | . | . | . | . | . | . | . | 1 | . | . | . | . | . | . | . | . | * |
| *Galium verum* | . | . | . | . | . | . | . | . | 1 | 1 | 0.1 | 2 | . | . | . | . | 1 | . | . | . | . | . | . | . | * |
| *Geranium molle* | . | . | . | . | . | . | . | . | . | . | . | . | . | . | . | . | 1 | . | . | . | . | . | . | . | * |
| *Geranium pusillum* | . | . | . | . | . | . | . | . | . | . | . | . | . | . | . | . | 0.1 | . | . | . | . | . | . | . | * |
| *Geum urbanum* | . | . | 0.1 | . | . | . | . | . | . | . | . | . | . | . | . | . | . | . | . | . | . | . | . | . | * |
| *Helichrysum arenarium* | . | . | . | . | . | 1 | 1 | . | 1 | . | . | . | . | . | . | . | . | . | . | . | . | 4 | X | . | * |
| *Herniaria glabra* | . | 1 | . | . | . | . | . | . | . | . | . | . | . | 1 | . | . | 0.1 | . | . | . | 0.1 | . | . | . | * |
| *Hieracium pilosella* | . | . | . | . | . | . | . | 1 | . | 1 | . | 20 | . | 1 | . | 20 | . | . | 4 | . | 1 | . | X | . | * |
| *Hieracium umbellatum* | . | . | . | 1 | . | . | . | . | . | . | . | . | . | . | . | . | . | . | . | . | . | . | . | . | * |
| *Holcus lanatus* | . | . | . | . | . | 1 | . | . | . | . | . | 2 | 1 | . | . | 2 | . | . | 2 | . | . | . | . | . | * |
| *Holcus mollis* | . | . | . | . | . | . | . | . | . | 2 | . | . | . | . | . | . | . | . | . | . | . | . | . | . | * |
| *Holosteum umbellatum* | . | . | . | . | . | . | . | . | . | . | . | . | . | . | . | . | . | . | . | 1 | . | 2 | X | . | V |
| *Hypericum perforatum* | . | 0.1 | . | 0.1 | 0.1 | . | . | . | . | 1 | . | . | 0.1 | 1 | . | . | . | 1 | 1 | . | . | . | . | . | * |
| *Hypochaeris radicata* | . | 0.1 | . | . | 1 | 0.1 | . | . | . | . | 0.1 | 0.1 | 1 | . | 1 | 0.1 | . | . | . | . | 0.1 | 0.1 | . | . | * |
| *Jasione montana* | . | . | . | . | . | . | . | 1 | . | . | . | . | . | 1 | . | . | . | . | 0.1 | . | 0.1 | 1 | X | . | * |
| *Lamium purpureum* | . | . | 1 | . | . | . | . | . | . | . | . | . | . | . | . | . | . | . | . | . | . | . | . | . | * |
| *Linaria vulgaris* | . | . | . | . | 1 | . | . | . | . | 1 | . | . | . | . | . | . | . | . | . | . | 1 | . | . | . | * |
| *Lolium perenne* | 4 | . | . | . | . | . | . | . | . | . | . | . | . | . | . | . | . | . | . | . | . | . | . | . | * |
| *Lotus corniculatus* | . | . | . | 0.1 | . | . | . | . | . | . | . | . | . | . | . | . | . | . | . | . | . | . | . | . | * |
| *Luzula campestris* | . | . | . | . | . | . | . | . | . | 1 | . | 30 | . | . | . | 10 | . | . | . | . | 2 | . | . | . | * |
| *Medicago lupulina* | 0.1 | . | . | 1 | . | . | . | . | . | 0.1 | . | . | . | . | . | . | . | . | . | . | . | . | . | . | * |
| *Medicago* x *varia* | . | . | . | . | 0.1 | . | . | . | . | . | . | . | . | . | . | . | . | . | . | . | 0.1 | . | . | X | * |
| *Melilotus albus* | . | 0.1 | . | . | . | . | . | . | . | . | . | . | . | . | . | . | . | . | . | . | . | . | . | . | * |
| *Melilotus officinalis* | . | . | . | 0.1 | . | . | . | . | . | . | . | . | . | . | . | . | . | . | . | . | . | . | . | . | * |
| *Melampyrum pratense* | . | . | . | . | . | . | . | . | . | 1 | . | . | . | . | . | . | . | . | . | . | . | . | . | . | * |
| *Myosotis arvensis* | . | . | . | . | . | . | . | . | . | 0.1 | . | . | . | . | . | . | . | . | . | . | . | . | . | . | * |
| *Myosotis ramosissima* | . | . | 2 | . | 1 | . | . | 1 | . | 0.1 | . | 1 | 1 | 1 | . | . | 1 | 1 | 1 | 1 | 2 | 1 | X | . | * |
| **Species** | **1** | **2** | **3** | **4** | **5** | **6** | **7** | **8** | **9** | **10** | **11** | **12** | **13** | **14** | **15** | **16** | **17** | **18** | **19** | **20** | **21** | **22** | **DGS** | **N** | **RL** |
| *Myosotis* spec. | . | . | . | 0.1 | . | . | . | . | . | . | . | . | . | . | . | . | . | . | . | . | . | . | . | . | * |
| *Myosotis stricta* | . | . | . | . | . | . | . | . | . | . | . | . | 1 | . | . | . | . | 1 | 0.1 | . | . | . | X | . | * |
| *Oenothera biennis* agg. | . | 0.1 | . | 1 | 1 | . | 0.1 | . | . | . | . | . | . | . | . | . | . | . | . | . | . | . | X | X | * |
| *Papaver dubium* | . | . | . | . | . | . | . | . | . | . | . | . | . | . | . | . | . | . | . | . | . | 0.1 | . | . | * |
| *Petrorhagia prolifera* | . | . | . | 1 | . | . | 1 | . | . | . | . | . | . | . | . | . | . | . | . | 1 | 1 | . | X | . | * |
| *Peucedanum oreoselinum* | 2 | . | 2 | . | . | . | . | . | . | 1 | . | . | . | . | . | 1 | . | . | . | . | . | . | X | . | 3 |
| *Pinus sylvestris* | . | . | . | . | . | . | . | 0.1 | . | . | . | . | . | 0.1 | . | . | . | . | . | . | . | . | . | . | * |
| *Plantago lanceolata* | 1 | 1 | 1 | 1 | . | . | . | . | . | 1 | 1 | 1 | . | . | 1 | . | 1 | . | . | 1 | 2 | . | . | . | * |
| *Plantago major* | . | . | 1 | . | . | . | . | . | . | . | . | . | . | . | . | . | . | . | . | . | . | . | . | . | * |
| *Poa angustifolia* | 10 | 1 | 20 | 1 | 20 | 1 | . | 1 | . | 10 | 20 | 4 | 10 | . | 2 | . | 20 | . | . | 1 | 20 | . | . | . | * |
| *Poa annua* | 0.1 | . | . | . | . | . | . | . | . | . | . | . | . | . | . | . | . | . | . | . | . | . | . | . | * |
| *Poa bulbosa* | . | . | . | . | . | . | . | . | . | . | . | . | . | . | . | . | . | . | . | 10 | . | . | . | . | * |
| *Poa compressa* | . | 1 | 1 | 1 | . | . | . | . | 2 | . | . | . | . | . | . | . | 1 | 1 | . | . | . | . | X | . | * |
| *Poa trivialis* | . | . | 2 | . | . | . | . | . | . | . | . | . | . | . | . | . | . | . | . | . | . | . | . | . | * |
| *Polygonatum odoratum* | . | . | 0.1 | . | . | . | . | . | . | . | . | . | . | . | . | . | . | . | . | . | . | . | . | . | V |
| *Polygonum aviculare* agg. | 1 | . | 0.1 | . | 0.1 | . | . | . | . | . | . | 0.1 | . | . | . | . | . | . | . | . | . | . | . | . | * |
| *Populus tremula* | . | . | . | . | . | . | . | . | . | 0.1 | . | . | . | . | . | . | . | . | . | . | . | . | . | . | * |
| *Potentilla argentea* | . | 0.1 | 2 | . | 1 | . | 1 | . | . | . | 2 | 2 | 0.1 | . | . | . | 1 | 1 | . | 1 | 1 | . | X | . | * |
| *Potentilla incana* | 1 | . | . | . | . | . | . | . | . | 0.1 | . | . | . | . | . | . | 2 | . | . | . | . | . | X | . | 2 |
| *Potentilla reptans* | . | . | . | . | . | 0.1 | . | . | . | . | . | . | . | . | . | . | . | . | . | . | . | . | . | . | * |
| *Potentilla verna* | . | . | . | . | . | . | . | . | 4 | . | . | . | . | . | . | . | . | . | . | 4 | . | . | X | . | * |
| *Prunus serotina* | . | . | . | . | . | . | . | . | . | 0.1 | . | . | . | . | . | . | 0.1 | . | . | . | . | . | . | X | * |
| *Prunus* spec. | . | . | . | . | 0.1 | . | . | . | . | . | . | . | . | . | . | . | . | . | . | . | . | . | . | NA | * |
| *Prunus spinosa* | . | . | . | . | . | . | . | . | . | . | . | . | . | . | . | . | . | . | 2 | . | . | . | . | . | * |
| *Quercus robur* | . | . | . | . | 0.1 | . | . | . | . | . | . | . | . | . | . | . | . | . | . | . | . | . | . | . | * |
| *Ranunculus acris* | . | . | . | . | . | . | . | . | . | . | . | . | 0.1 | . | . | . | . | . | . | . | . | . | . | . | * |
| *Ranunculus bulbosus* | . | . | . | . | . | . | . | . | . | . | 10 | . | . | . | . | . | . | . | . | . | . | . | X | . | 3 |
| *Robinia pseudoacacia* | . | . | 0.1 | . | . | . | . | . | . | . | . | . | . | . | . | . | 0.1 | . | . | . | . | . | . | X | * |
| *Rubus caesius* | 4 | . | . | . | . | . | 1 | . | . | 1 | . | . | . | . | . | . | . | . | . | . | . | . | . | . | * |
| *Rumex acetosella* | . | 0.1 | . | . | 1 | 0.1 | 0.1 | 1 | 1 | . | 1 | 1 | 2 | 1 | 2 | 1 | 1 | 1 | 2 | . | . | 1 | X | . | * |
| **Species** | **1** | **2** | **3** | **4** | **5** | **6** | **7** | **8** | **9** | **10** | **11** | **12** | **13** | **14** | **15** | **16** | **17** | **18** | **19** | **20** | **21** | **22** | **DGS** | **N** | **RL** |
| *Rumex thyrsiflorus* | 0.1 | . | 1 | 0.1 | 10 | 0.1 | . | . | . | . | 4 | 0.1 | 0.1 | . | 1 | . | . | . | . | . | 2 | . | . | . | * |
| *Saponaria officinalis* | 1 | . | 0.1 | 1 | . | . | . | . | . | . | . | . | . | . | . | . | . | . | . | . | . | . | . | . | * |
| *Scleranthus annuus* agg. | . | 0.1 | . | . | . | . | . | . | . | . | . | . | . | . | 0.1 | . | . | . | . | . | . | . | X | . | * |
| *Scorzoneroides autumnalis* | . | . | . | . | . | . | . | . | . | . | . | 0.1 | . | . | . | . | . | . | . | . | . | . | . | . | * |
| *Sedum acre* | . | . | . | . | . | . | 0.1 | . | . | . | . | . | . | . | . | . | . | . | . | . | . | 1 | X | . | * |
| *Sedum sexangulare* | . | . | . | . | . | . | . | . | 2 | . | . | . | . | . | . | . | . | . | . | 0.1 | . | . | X | . | * |
| *Senecio inaequidens* | . | . | . | . | . | . | . | . | . | . | . | . | . | . | . | . | . | . | . | . | . | 0.1 | . | X | * |
| *Senecio jacobaea* | . | . | . | 0.1 | . | . | . | . | . | 0.1 | . | . | . | 0.1 | . | . | . | . | . | . | . | . | . | . | * |
| *Senecio vernalis* | . | . | . | . | . | . | . | . | . | . | . | . | . | . | . | . | . | . | . | . | . | 1 | . | X | * |
| *Setaria viridis* | 2 | . | . | . | . | 1 | 1 | 1 | 2 | . | . | 0.1 | . | . | . | . | 1 | . | . | 2 | . | . | . | . | * |
| *Silene latifolia* subsp. *alba* | . | . | 0.1 | 0.1 | 1 | . | . | . | . | 0.1 | . | . | . | . | . | . | . | . | . | . | . | . | . | . | * |
| *Solidago canadensis* | . | 1 | . | 1 | . | . | . | . | . | . | . | . | . | . | . | 0.1 | . | . | . | . | . | . | . | X | * |
| *Spergula morisonii* | . | . | . | . | . | . | . | . | . | . | . | . | . | 1 | . | . | . | . | . | . | . | . | X | . | * |
| *Stellaria graminea* | . | . | . | . | . | . | . | . | . | . | . | 1 | 1 | . | . | . | . | . | . | . | . | . | . | . | * |
| *Stellaria media* | . | . | 0.1 | . | . | . | . | . | . | . | . | . | . | . | . | . | . | . | . | . | . | . | . | . | * |
| *Stellaria pallida* | . | . | 0.1 | . | 0.1 | . | . | . | . | . | . | . | . | . | 1 | . | . | . | . | . | . | . | . | . | * |
| *Tanacetum vulgare* | . | . | . | 0.1 | . | . | . | . | . | . | . | 1 | . | 4 | . | . | . | 1 | . | . | 2 | . | . | . | * |
| *Taraxacum* sect. *Erythrosperma* | . | . | . | . | . | . | . | . | . | 1 | . | 1 | . | . | . | . | . | . | . | . | . | . | . | . | * |
| *Taraxacum* sect. *Ruderalia* | 1 | . | 0.1 | . | 0.1 | . | . | . | . | 0.1 | . | 0.1 | . | . | . | . | 0.1 | . | . | . | . | . | . | . | * |
| *Teesdalia nudicaulis* | . | . | . | . | . | . | . | . | . | . | . | . | . | . | . | . | . | 4 | . | . | . | . | X | . | V |
| *Thymus pulegioides* | . | . | . | . | . | . | . | . | . | . | . | 1 | . | . | . | 10 | . | . | . | . | . | . | X | . | V |
| *Tragopogon dubius* | . | . | . | . | 0.1 | . | . | . | . | . | 1 | . | . | . | . | . | . | . | . | . | . | . | . | . | * |
| *Tragopogon* spec. | . | . | . | . | . | . | . | . | . | . | . | . | . | . | . | . | . | . | . | . | 0.1 | . | . | . | * |
| *Trifolium arvense* | . | 1 | . | . | 1 | . | 0.1 | . | 0.1 | . | 0.1 | 0.1 | . | . | 0.1 | . | . | 0.1 | . | 1 | 1 | 1 | X | . | * |
| *Trifolium campestre* | . | 0.1 | . | 1 | . | . | . | . | . | . | . | . | . | . | . | . | . | . | . | . | 1 | . | . | . | * |
| *Trifolium dubium* | . | . | . | . | . | . | . | . | . | . | . | . | . | . | 0.1 | . | . | . | . | . | . | . | . | . | * |
| *Trifolium pratense* | . | 0.1 | . | . | . | . | . | . | . | . | . | . | . | . | . | . | . | . | . | . | . | . | . | . | * |
| *Trifolium repens* | 0.1 | 1 | 0.1 | . | . | . | . | . | . | . | . | . | . | . | . | . | . | . | . | . | . | . | . | . | * |
| *Turritis glabra* | . | . | . | . | 1 | . | . | . | . | . | . | 0.1 | . | . | . | . | . | 0.1 | . | . | . | . | X | . | * |
| *Verbascum lychnitis* | . | . | . | 0.1 | 2 | . | . | . | . | . | . | . | . | . | 1 | . | . | 0.1 | . | . | . | . | . | . | * |
| **Species** | **1** | **2** | **3** | **4** | **5** | **6** | **7** | **8** | **9** | **10** | **11** | **12** | **13** | **14** | **15** | **16** | **17** | **18** | **19** | **20** | **21** | **22** | **DGS** | **N** | **RL** |
| *Verbascum nigrum* | . | . | . | . | . | . | 0.1 | . | . | . | . | . | . | . | . | . | . | . | . | . | 0.1 | . | . | . | * |
| *Verbascum thapsus* | . | . | . | 0.1 | . | . | . | . | . | . | . | . | . | . | . | . | . | . | . | . | . | . | . | . | * |
| *Verbena officinalis* | . | . | . | . | . | . | . | . | . | . | . | . | . | . | . | . | . | . | . | . | 1 | . | . | . | * |
| *Veronica arvensis* | 1 | 1 | 2 | . | . | . | . | 1 | 0.1 | . | 1 | 1 | 1 | . | 2 | . | 1 | 1 | 1 | 1 | 1 | 1 | . | . | * |
| *Veronica chamaedrys* | . | . | 1 | . | . | . | . | . | . | . | . | 1 | 1 | . | . | . | 1 | . | . | . | . | . | . | . | * |
| *Veronica prostrata* | . | . | . | . | . | . | . | . | . | . | 2 | . | . | . | . | . | . | . | . | . | . | . | X | . | 2 |
| *Veronica sublobata* | . | . | 1 | . | . | . | . | . | . | . | . | . | . | . | . | . | . | . | . | . | . | . | . | . | * |
| *Veronica verna* | . | . | . | . | . | . | . | . | . | . | . | . | . | . | 0.1 | . | . | . | . | . | . | . | X | . | 2 |
| *Vicia angustifolia* | 1 | . | 1 | 1 | . | 0.1 | 0.1 | . | . | 1 | . | . | 1 | . | . | 0.1 | 0.1 | . | . | . | 0.1 | . | . | . | * |
| *Vicia cassubica* | . | . | . | . | . | . | . | . | . | 1 | . | . | . | . | . | . | . | . | . | . | . | . | . | . | 3 |
| *Vicia hirsuta* | . | 0.1 | 1 | . | 0.1 | . | 0.1 | . | . | . | . | . | . | . | . | . | . | 0.1 | . | . | 0.1 | 1 | . | . | * |
| *Vicia lathyroides* | 0.1 | . | 0.1 | . | 1 | 1 | 1 | . | . | . | . | . | . | . | . | . | 1 | . | . | 1 | 0.1 | . | X | . | * |
| *Vicia tetrasperma* | . | . | . | . | . | . | . | . | . | 0.1 | . | . | . | . | . | . | . | . | . | . | . | . | . | . | * |
| *Viola arvensis* | . | . | 0.1 | . | . | . | . | . | . | . | . | . | . | . | . | . | 0.1 | . | . | . | . | . | . | . | * |
| *Viola canina* | . | . | . | . | . | . | . | . | . | 0.1 | . | 0.1 | . | . | . | 1 | . | . | 0.1 | . | . | . | . | . | V |
| *Viola suavis* | . | . | . | . | 2 | . | . | . | . | . | . | . | . | . | . | . | . | . | . | . | . | . | . | X | * |

| **Table S3** Abundances of grasshoppers recorded in September 2019 and July 2020 and their status as Red List species and dry grassland specialist. DGS = Dry grassland specialist, RL = Red List categories are according to (Machatzi et al. (2005): 0 = extinct, 1 = threatened of extinction, 3 = vulnerable, V = near threatened, *= not endangered). We identified grasshopper species according to Wendler et al. (1999) and Bellmann (2006). For acoustical assessment, we used audio files by Garberding (2001) and (Bellmann, 2004). | | | | | | | | | | | | | | | | | | | | | | | | |
| --- | --- | --- | --- | --- | --- | --- | --- | --- | --- | --- | --- | --- | --- | --- | --- | --- | --- | --- | --- | --- | --- | --- | --- | --- |
| **Species** | **1** | **2** | **3** | **4** | **5** | **6** | **7** | **8** | **9** | **10** | **11** | **12** | **13** | **14** | **15** | **16** | **17** | **18** | **19** | **20** | **21** | **22** | **DGS** | **RL** |
| *Bicolorana bicolor* | . | . | 1 | 1 | . | 3 | 2 | . | . | . | 10 | . | . | . | 9 | . | . | 1 | . | . | 4 | 1 | X | V |
| *Calliptamus italicus* | . | 5 | . | 2 | . | . | 1 | . | . | . | 4 | . | . | 3 | 2 | 3 | 4 | 2 | . | . | 8 | . | X | 0 |
| *Chorthippus albomarginatus* | . | . | . | . | . | . | . | . | . | . | . | . | 5 | . | . | . | . | . | . | . | . | . | . | * |
| *Chorthippus apricarius* | 4 | . | 2 | 6 | . | . | . | 1 | . | . | . | 1 | 1 | 2 | 1 | . | 3 | . | . | . | 3 | . | . | * |
| *Chorthippus biguttulus* | 37 | . | 10 | 17 | 4 | 45 | 16 | . | 1 | 38 | 33 | 63 | 3 | 8 | 9 | 25 | 61 | 39 | 37 | 10 | 39 | . | . | * |
| *Chorthippus brunneus* | 7 | 9 | 5 | 8 | 34 | 53 | 15 | 32 | . | 12 | 38 | 12 | 23 | 32 | 31 | 8 | 6 | 43 | 22 | 23 | 61 | 13 | X | * |
| *Chorthippus dorsatus* | 2 | . | . | 14 | 3 | . | 2 | 1 | . | . | 24 | 33 | 14 | 2 | . | 2 | 1 | . | . | . | 12 | 8 | . | * |
| *Chorthippus mollis* | 34 | 37 | 6 | 35 | 15 | 32 | 36 | 8 | 14 | . | 42 | 27 | 31 | 13 | 36 | 26 | 14 | 15 | 17 | 42 | 41 | 63 | X | * |
| *Chrysochraon dispar* | . | . | . | . | . | . | 5 | . | 1 | . | . | . | . | . | . | . | . | . | . | . | 1 | . | . | * |
| *Conocephalus dorsalis* | . | . | . | . | . | . | . | 1 | . | . | 2 | . | 8 | . | . | 4 | . | . | . | . | 1 | . | . | V |
| *Conocephalus fuscus* | 2 | . | . | . | . | . | 12 | 1 | . | . | 4 | 3 | . | 1 | . | 5 | . | . | . | . | 3 | . | . | * |
| *Decticus verrucivorus* | . | . | . | . | . | . | 3 | . | . | . | 1 | . | . | . | . | 14 | . | . | . | . | . | . | X | 1 |
| *Gryllus campestris* | . | . | . | . | . | . | . | . | . | . | . | . | . | . | . | 6 | . | . | . | . | . | . | X | 1 |
| *Leptophyes punctatissima* | 2 | . | 3 | . | . | . | 1 | . | . | . | . | 1 | . | 1 | . | . | 4 | 1 | . | 1 | 1 | . | . | * |
| *Maconema thalassinum* | . | . | . | . | . | . | . | . | . | 1 | . | . | . | . | . | . | . | . | . | . | . | . | . | * |
| *Myrmeleotettix maculatus* | 1 | 11 | . | 3 | . | . | 20 | 10 | 1 | . | 2 | . | . | 9 | 3 | . | 1 | . | . | . | 1 | . | X | V |
| *Oedipoda caerulescens* | 1 | 2 | . | . | . | 4 | 2 | 3 | 1 | . | 5 | . | 2 | 2 | 4 | 6 | . | 5 | 1 | 10 | 8 | 1 | X | V |
| *Omocestus haemorrhoidalis* | . | . | . | 1 | . | . | . | . | . | . | . | . | . | . | . | . | . | . | . | . | . | . | X | V |
| *Phaneroptera falcata* | . | . | . | . | 1 | . | 1 | . | . | . | 1 | . | . | . | . | 1 | . | . | . | . | . | . | . | * |
| *Platycleis albopunctata* | . | 2 | . | 1 | . | 2 | 5 | 1 | 1 | . | 1 | 2 | . | 2 | 2 | 2 | . | 3 | 2 | 4 | 12 | 4 | X | V |
| *Pseudochorthippus parallelus* | 1 | . | 5 | 8 | 7 | 1 | 5 | 6 | 1 | . | 22 | 22 | 6 | 4 | 5 | 16 | 11 | . | 15 | 2 | 16 | . | . | * |
| *Roeseliana roeselii* | 1 | . | 8 | 5 | . | . | . | . | . | . | . | 2 | 9 | . | . | . | . | . | . | . | . | . | . | * |
| *Stenobothrus lineatus* | . | . | . | 2 | . | 1 | 9 | 4 | . | . | 10 | 10 | . | . | 1 | 13 | 1 | 2 | 3 | . | 5 | . | X | 3 |
| *Tettigonia viridissima* | . | 2 | . | 4 | . | 3 | . | 1 | 2 | . | 1 | . | 3 | 2 | . | . | . | . | 2 | . | . | . | . | * |

| **Table S4** Abundances of sand lizards. Counts are displayed for period 1(May 2020) and period 2 (June 2020) on each study site. Summed counts are given in column "sum". | | | |
| --- | --- | --- | --- |
| **Study site** | **Period 1** | **Period 2** | **Sum** |
| **1** | 2 | 0 | 2 |
| **2** | 5 | 1 | 6 |
| **3** | 3 | 0 | 3 |
| **4** | 0 | 0 | 0 |
| **5** | 0 | 1 | 1 |
| **6** | 0 | 1 | 1 |
| **7** | 6 | 7 | 13 |
| **8** | 9 | 4 | 13 |
| **9** | 0 | 0 | 0 |
| **10** | 1 | 1 | 2 |
| **11** | 0 | 1 | 1 |
| **12** | 0 | 2 | 2 |
| **13** | 0 | 0 | 0 |
| **14** | 5 | 4 | 9 |
| **15** | 0 | 5 | 5 |
| **16** | 6 | 1 | 7 |
| **17** | 0 | 0 | 0 |
| **18** | 0 | 0 | 0 |
| **19** | 0 | 0 | 0 |
| **20** | 3 | 1 | 4 |
| **21** | 1 | 2 | 3 |
| **22** | 0 | 0 | 0 |

| **Table S5** Comparison of AIC values. Values were separately obtained for GLMMs calculated with rooting data as predictor (models rooting) and with frequencies (models frequencies). | | | |
| --- | --- | --- | --- |
| **Response variables** | **GLMM Family** | **AIC "models frequencies"** | **AIC "models rooting"** |
|  |  |  |  |
| ***Vascular plants*** |  |  |  |
| Species richness | Gaussian | 141.0 | 138.2 |
| Simpson diversity | Gaussian | -24.4 | -30.3 |
| Dry grassland species richness | Gaussian | 85.7 | 85.9 |
| Dry grassland specialists relative abundance | Negative-binomial | 40.2 | 39.5 |
| Red List species richness | Gaussian | 85.9 | 85.3 |
| Red List species relative abundance | Negative-binomial | 17.7 | 17.6 |
| Neophytes species richness | Gaussian | 81.3 | 81.4 |
| Neophytes species relative abundance | Negative-binomial | 17.9 | 17.9 |
| ***Grasshoppers*** |  |  |  |
| Species richness | Gaussian | 105.9 | 104.6 |
| Simpson diversity | Gaussian | -7.4 | -8.2 |
| Dry grassland species richness | Gaussian | 90.2 | 87.0 |
| Dry grassland specialists relative abundance | Negative-binomial | 42.4 | 42.8 |
| Red List species richness | Gaussian | 90.0 | 88.1 |
| Red List species relative abundance | Negative-binomial | 25.2 | 25.1 |
| ***Sand lizards*** |  |  |  |
| Abundance | Poisson | 105.3 | 103.6 |

| **Table S6** GLMM summaries. Wild boar activity indices and their predicted effect on vascular plant and grasshopper communities as well as sand lizard populations using generalised linear mixed models (GLMM). The column “GLMM Family” informs about the GLMM type used. The estimates (Est.), standard errors (SE), t-values (t), z-values (z) and p-values (p) are displayed for fixed variables. Significant fixed variables (p < 0.05) are indicated in bold. The variance (Var.) and the standard deviation (Std. dev.) are displayed for the random variables. The Goodness-of-fit is indicated by R². | | | | | | | | | |
| --- | --- | --- | --- | --- | --- | --- | --- | --- | --- |
|  | **GLMM Family** | **Fixed Variables** | | | **Random variables** | | | | **R²** |
|  |  |  | **Proportion of**  **fresh rooting** | **Proportion of old rooting** |  | **Cover of herbs** | **Cover of litter** | **Maximum height of herbs** |  |
| **Vascular plants** |  |  |  |  |  |  |  |  |  |
| Species richness | Gaussian | **Est.** | 2.1934 | 0.0095 | **Var.** | 16.7100 | 24.2600 | 0.0000 | 0.484 |
|  |  | **SE** | 1.6424 | 1.6767 | **Std. dev.** | 4.0880 | 4.9260 | 0.0000 |  |
|  |  | ***t*** | 1.3360 | 0.0060 |  |  |  |  |  |
|  |  | ***p*** | 0.2010 | 0.9960 |  |  |  |  |  |
| Simpson diversity | Gaussian | **Est.** | 0.0077 | **-0.0538** | **Var.** | 0.0000 | 0.0013 | 0.0001 | 0.649 |
|  |  | **SE** | 0.0114 | **0.0114** | **Std. dev.** | 0.0000 | 0.0360 | 0.0083 |  |
|  |  | ***t*** | 0.6770 | **-4.7310** |  |  |  |  |  |
|  |  | ***p*** | 0.5085 | **0.0002** |  |  |  |  |  |
| Dry grassland species richness | Gaussian | **Est.** | 0.6490 | -0.1932 | **Var.** | 0.0000 | 0.0000 | 0.0000 | 0.060 |
|  |  | **SE** | 0.5912 | 0.5912 | **Std. dev.** | 0.0000 | 0.0000 | 0.0000 |  |
|  |  | ***t*** | 1.098 | -0.3270 |  |  |  |  |  |
|  |  | ***p*** | 0.2860 | 0.7470 |  |  |  |  |  |
| Dry grassland specialists relative abundance | Neg.-binomial | **Est.** | -0.0592 | 0.1881 | **Var.** | 0.0000 | 0.0000 | 0.0000 | 0.172 |
|  |  | **SE** | 0.2954 | 0.2696 | **Std. dev.** | 0.0000 | 0.0000 | 0.0000 |  |
|  |  | ***z*** | -0.2000 | 0.6970 |  |  |  |  |  |
|  |  | ***p*** | 0.8411 | 0.4855 |  |  |  |  |  |
| Red List species richness | Gaussian | **Est.** | -0.0947 | -0.6458 | **Var.** | 0.0000 | 0.0000 | 0.0000 | 0.140 |
|  |  | **SE** | 0.3854 | 0.3854 | **Std. dev.** | 0.0000 | 0.0000 | 0.0000 |  |
|  |  | ***t*** | -0.2460 | -1.6760 |  |  |  |  |  |
|  |  | ***p*** | 0.8085 | 0.1102 |  |  |  |  |  |
| Red List species relative abundance | Neg. -binomial | **Est.** | -0.2280 | -0.3696 | **Var.** | 0.0000 | 0.0000 | 0.0000 | 0.150 |
|  |  | **SE** | 1.0505 | 1.1573 | **Std. dev.** | 0.0000 | 0.0000 | 0.0000 |  |
|  |  | ***z*** | -0.2170 | -0.3190 |  |  |  |  |  |
|  |  | ***p*** | 0.8282 | 0.7494 |  |  |  |  |  |
| Neophytes species richness | Gaussian | **Est.** | -0.0368 | 0.1155 | **Var.** | 0.1085 | 0.0000 | 0.0000 | 0.045 |
|  |  | **SE** | 0.3414 | 0.3436 | **Std. dev.** | 0.3294 | 0.0000 | 0.0000 |  |
|  |  | ***t*** | -0.1080 | 0.3360 |  |  |  |  |  |
|  |  | ***p*** | 0.9154 | 0.7406 |  |  |  |  |  |
| Neophytes species relative abundance | Neg.-binomial | **Est.** | 0.0320 | 0.0642 | **Var.** | 0.0000 | 0.0000 | 0.0000 | 0.002 |
|  |  | **SE** | 1.0683 | 1.0599 | **Std. dev.** | 0.0000 | 0.0000 | 0.0000 |  |
|  |  | ***z*** | 0.0300 | 0.0610 |  |  |  |  |  |
|  |  | ***p*** | 0.9761 | 0.9517 |  |  |  |  |  |
|  | **GLMM Family** | **Fixed Variables** | | | **Random variables** | | | | **R²** |
|  |  |  | **Proportion of**  **fresh rooting** | **Proportion of**  **old rooting** |  | **Cover of herbes** | **Cover of**  **litter** | **Maximum height of herbes** |  |
| **Grasshoppers** |  |  |  |  |  |  |  |  |  |
| Species richness | Gaussian | **Est.** | **-1.0969** | **1.8894** | **Var.** | 8.2820 | 0.0000 | 2.6320 | 0.812 |
|  |  | **SE** | **0.4613** | **0.5315** | **Std. dev.** | 2.8780 | 0.0000 | 1.6220 |  |
|  |  | ***t*** | **-2.3780** | **3.5550** |  |  |  |  |  |
|  |  | ***p*** | **0.0478** | **0.0048** |  |  |  |  |  |
| Simpson diversity | Gaussian | **Est.** | **-0.0450** | **0.0817** | **Var.** | 0.0099 | 0.0000 | 0.0004 | 0.692 |
|  |  | **SE** | **0.0190** | **0.0211** | **Std. dev.** | 0.0993 | 0.0000 | 0.0199 |  |
|  |  | ***t*** | **-2.3700** | **3.8640** |  |  |  |  |  |
|  |  | ***p*** | **0.0370** | **0.0016** |  |  |  |  |  |
| Dry grassland species richness | Gaussian | **Est.** | -0.6217 | **1.0098** | **Var.** | 0.0000 | 0.0000 | 1.3270 | 0.513 |
|  |  | **SE** | 0.3759 | **0.3960** | **Std. dev.** | 0.0000 | 0.0000 | 1.1520 |  |
|  |  | ***t*** | -1.6540 | **2.5500** |  |  |  |  |  |
|  |  | ***p*** | 0.1173 | **0.0202** |  |  |  |  |  |
| Dry grassland specialists relative abundance | Neg. -binomial | **Est.** | -0.0988 | 0.1159 | **Var.** | 0.0000 | 0.0000 | 0.0000 | 0.155 |
|  |  | **SE** | 0.2810 | 0.2553 | **Std. dev.** | 0.0000 | 0.0000 | 0.0000 |  |
|  |  | ***z*** | -0.3510 | 0.4540 |  |  |  |  |  |
|  |  | ***p*** | 0.7253 | 0.6499 |  |  |  |  |  |
| Red List species richness | Gaussian | **Est.** | -0.5762 | **0.9588** | **Var.** | 0.0000 | 0.0000 | 1.4450 | 0.477 |
|  |  | **SE** | 0.4018 | **0.4228** | **Std. dev.** | 0.0000 | 0.0000 | 1.2020 |  |
|  |  | ***t*** | -1.4340 | **2.2680** |  |  |  |  |  |
|  |  | ***p*** | 0.1709 | **0.0361** |  |  |  |  |  |
| Red List species relative abundance | Neg. -binomial | **Est.** | -0.1476 | 0.3817 | **Var.** | 0.0000 | 0.0000 | 0.0000 | 0.279 |
|  |  | **SE** | 0.6154 | 0.5472 | **Std. dev.** | 0.0000 | 0.0000 | 0.0000 |  |
|  |  | ***z*** | -0.2400 | 0.6980 |  |  |  |  |  |
|  |  | ***p*** | 0.8104 | 0.4854 |  |  |  |  |  |
| **Sand lizards** |  |  |  |  |  |  |  |  |  |
| Abundance | Poisson | **Est.** | -0.4419 | **0.7326** | **Var.** | 0.3273 | 0.4107 | 0.8511 | 0.796 |
|  |  | **SE** | 0.3161 | **0.2682** | **Std. dev.** | 0.5721 | 0.6409 | 0.9225 |  |
|  |  | ***z*** | -1.398 | **2.7310** |  |  |  |  |  |
|  |  | ***p*** | 0.16212 | **0.0063** |  |  |  |  |  |

| **Tables S7** Species scores obtained by partial-RDA applied on grasshopper data. The column RDA1 indicates the scores obtained along the constrained axe describing the proportion of old rooting. The column PC1 indicates the scores obtained along the first axe in principal component. | | | |
| --- | --- | --- | --- |
| Species code | Species name | RDA1 | PC1 |
| Myr.mac | *Myrmeleotettix maculatus* | 0.821069 | -0.06342 |
| Cal.ita | *Calliptamus italicus* | 0.366787 | 0.37243 |
| Tet.vir | *Tettigonia viridissima* | 0.340813 | -0.07037 |
| Pla.alb | *Platycleis albopunctata* | 0.278096 | 0.47764 |
| Cho.bru | *Chorthippus brunneus* | 0.223518 | 0.53762 |
| Cho.mol | *Chorthippus mollis* | 0.215186 | 0.38537 |
| Chr.dis | *Chrysochraon dispar* | 0.192885 | 0.08362 |
| Oed.cae | *Oedipoda caerulescens* | 0.175212 | 0.49969 |
| Con.fus | *Conocephalus fuscus* | 0.146769 | 0.385 |
| Bic.bic | *Bicolorana bicolor* | 0.137917 | 0.38456 |
| Cho.apr | *Chorthippus apricarius* | 0.121983 | -0.07123 |
| Cho.dor | *Chorthippus dorsatus* | 0.110415 | 0.18123 |
| Roe.roe | *Roeseliana roeselii* | 0.063499 | -0.44291 |
| Ste.lin | *Stenobothrus lineatus* | 0.041361 | 0.60389 |
| Con.dor | *Conocephalus dorsalis* | -0.001099 | 0.06359 |
| Pha.fal | *Phaneroptera falcata* | -0.016232 | 0.08021 |
| Pse.par | *Pseudochorthippus parallelus* | -0.035593 | 0.39495 |
| Dec.ver | *Decticus verrucivorus* | -0.044053 | 0.24704 |
| Lep.pun | *Leptophyes punctatissima* | -0.063719 | -0.02172 |
| Cho.big | *Chorthippus biguttulus* | -0.314461 | 0.8411 |

**References**

Bellmann, H. (2004). Heuschrecken. Die Stimmen von 61 heimischen Arten. (AMPLE Edition. Musikverlag, Germering.).

Bellmann, H. (2006). Der Kosmos Heuschreckenführer. Die Arten Mitteleuropas sicher bestimmen. (Kosmos, Stuttgart, Germany.).

Buttler, K.P., and Thieme, M. (2017). Florenliste von Deutschland, Version 9.

Garberding, K.H. (2001). Gesänge der heimischen Heuschrecken. Akustisch-optische Bestimmungshilfe. (Deutscher Jugendbund für Naturbeobachtung, Hamburg, Germany.).

Jäger, E.J. (Hrsg.) (2016). Rothmaler - Exkursionsflora von Deutschland: Gefäßpflanzen, Grundband, 21. Auflage, Heidelberg.

Machatzi, B., Ratsch, A., Prasse, R., and Ristow, M. (2005). Rote Liste und Gesamtartenliste der Heuschrecken und Grillen (Saltatoria: Ensifera et Caelifera) von Berlin.

Wendler, A., Lorenz, C., and Horstkotte, J. (1999). Heuschrecken Bestimmung, Verbreitung, Lebensräume und Gefährdung aller in Deutschland vorkommenden Arten. (Deutscher Jugendbund für Naturbeobachtung, Hamburg, Germany.).
